# One Step Forward towards the Development of Eco-Friendly Antifouling Coatings: Immobilization of a Sulfated Marine-Inspired Compound

**DOI:** 10.3390/md18100489

**Published:** 2020-09-25

**Authors:** Cátia Vilas-Boas, Francisca Carvalhal, Beatriz Pereira, Sílvia Carvalho, Emília Sousa, Madalena M. M. Pinto, Maria José Calhorda, Vitor Vasconcelos, Joana R. Almeida, Elisabete R. Silva, Marta Correia-da-Silva

**Affiliations:** 1 Laboratório de Química Orgânica e Farmacêutica, Departamento de Ciências Químicas, Faculdade de Farmácia, Universidade do Porto, R. Jorge de Viterbo Ferreira 228, 4050-313 Porto, Portugal; catiaboas94@gmail.com (C.V.-B.); francisca.carvalhal@gmail.com (F.C.); esousa@ff.up.pt (E.S.); madalena@ff.up.pt (M.M.M.P.); 2 CIIMAR—Centro Interdisciplinar de Investigação Marinha e Ambiental, Avenida General Norton de Matos, S/N, 4450-208 Matosinhos, Portugal; vmvascon@fc.up.pt (V.V.); joana.reis.almeida@gmail.com (J.R.A.); 3 BioISI—Instituto de Biosistemas e Ciências Integrativas, Departamento de Química e Bioquímica, Faculdade de Ciências, Universidade de Lisboa, Campo Grande, Lisboa, 1749-016 Portugal; beatrizmmgpereira@gmail.com (B.P.); mjcalhorda@fc.ul.pt (M.J.C.); 4 CQB—Centro de Química e Bioquímica, Departamento de Química e Bioquímica, Faculdade de Ciências, Universidade de Lisboa, Campo Grande, Lisboa, 1749-016 Lisboa, Portugal; scfcarvalho@fc.ul.pt; 5 Departamento de Biologia, Faculdade de Ciências, Universidade do Porto, Rua do Campo Alegre S/N, 4169-007 Porto, Portugal

**Keywords:** biofouling, marine coatings, anti-settlement, chemical synthesis, sulfated, gallic acid, eco-friendly

## Abstract

Marine biofouling represents a global economic and ecological challenge and few eco-friendly antifouling agents are available. The aim of this work was to establish the proof of concept that a recently synthesized nature-inspired compound (gallic acid persulfate, GAP) can act as an eco-friendly and effective antifoulant when immobilized in coatings through a non-release strategy, promoting a long-lasting antifouling effect. The synthesis of GAP was optimized to provide quantitative yields. GAP water solubility was assessed, showing values higher than 1000 mg/mL. GAP was found to be stable in sterilized natural seawater with a half-life (DT_50_) of 7 months. GAP was immobilized into several commercial coatings, exhibiting high compatibility with different polymeric matrices. Leaching assays of polydimethylsiloxane and polyurethane-based marine coatings containing GAP confirmed that the chemical immobilization of GAP was successful, since releases up to fivefold lower than the conventional releasing systems of polyurethane-based marine coatings were observed. Furthermore, coatings containing immobilized GAP exhibited the most auspicious anti-settlement effect against *Mytilus galloprovincialis* larvae for the maximum exposure period (40 h) in laboratory trials. Overall, GAP promises to be an agent capable of improving the antifouling activity of several commercial marine coatings with desirable environmental properties.

## 1. Introduction

Marine biofouling consists of the settlement and gradual accumulation of micro- and macro-organisms, such as bacteria, fungi, spores of algae, and invertebrate larvae in water submerged surfaces [1,2,3]. However, despite this natural process starting with the attachment and proliferation of bacteria, it is the growth of macrofouling organisms that most concerns marine industries. Their dense layers can cause a reduction in or even blockage of water flow in pipes, mechanical damage, corrosion, and the failure of equipment [4]. In other words, marine biofouling can increase maintenance costs and fuel consumption [5,6].

Chemical control is the principal strategy to combat this issue, combining traditional antifouling (AF) coatings, as is the case of polydimethylsiloxane (PDMS) and polyurethane (PU)-based coatings, with biocides, which are released over time [7,8]. Although most of them are presented as non-persistent biocides, several occurrence studies have concluded that, in fact, booster biocides persist, owing to their high release in biocide-release-based AF coatings [9,10,11,12]. In addition, due to their low water solubility and hydrophobic behavior, booster biocides tend to bioaccumulate, causing environmental damage [13,14,15]. Fortunately, there is increasing concern about the influence of copper and booster biocides in the marine environment and the effort to find ecological alternatives has led many researchers to develop greener AF approaches in order to reduce biocide release and consequently their persistence in the ecosystem [16,17,18,19,20]. A good AF agent must prevent fouling without persisting at concentrations greater than those that can cause detrimental effects to the environment.

**Figure 1:**
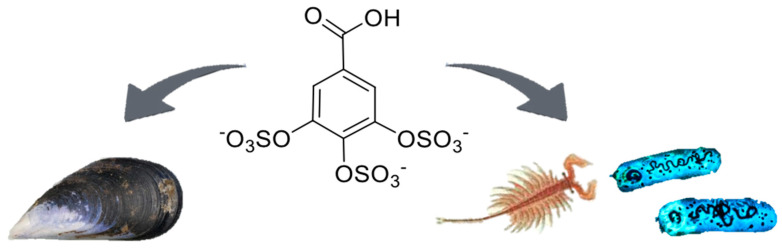
Antifouling (AF) activity and ecotoxicity previously discovered for gallic acid persulfate (GAP) [24].

A large variety of microorganisms and sessile marine organisms, such as sponges, corals, and algae, usually free of fouling on their surfaces, has been described to produce secondary metabolites to fight this natural process [21], as is the case of zosteric acid (ZA), a sulfated phenolic acid produced by the seagrass *Zostera marina* [22,23]. Inspired by the ZA structure, AF properties of a small library of synthetic sulfated small molecules were studied and gallic acid persulfate (GAP) was found to be one of the most promising compounds [24]. Furthermore, gallic acid, its starting material, is commercially available and easily accessible since it can be obtained from several sources, such as winery waste [25]. In contrast to ZA, GAP showed anti-settlement activity against the adhesive larvae of *Mytilus galloprovincialis*, one of the most aggressive invasive species in the world, according to the International Union for Conservation of Nature (IUCN) [26], without causing ecotoxicity against this species and other non-target species (Figure 1) [24].

To assess GAP suitability as an AF agent, it is necessary to evaluate compound stability and degradation pathways, optimize its large-scale synthesis, and analyze its behavior after incorporation in selected coating formulations. In this study, both the solubility of GAP in ultra-pure water (UPW) and in sterilized natural seawater (snSW), as well as the stability of sulfate groups after exposure to varying conditions of light and temperature for a period of several months, were examined. GAP synthesis was optimized through a microwave (MW) reaction to obtain high amounts of the compound and allow further immobilization in traditional marine coatings. Coating formulations with new synthesized GAP were developed using both direct incorporation (DI) and chemical immobilization (CI) strategies into two representative marine coatings: PDMS and PU-based coatings. The purpose of the chemical immobilization of GAP was to achieve maximal compatibility between this polar molecule and polymeric coatings, as well as provide long-lasting antifouling effects with a non-release strategy. Leaching assays were performed to confirm the minimal release of GAP into the aquatic environment. As the targets of any preventive technology are the colonizing stages [27], the settlement inhibition of *Mytilus galloprovincialis* larvae on the several GAP-based coating formulations was evaluated.

During these studies, two analytical methods were developed in order to: (1) directly analyze this sulfated polar compound in snSW, through an ion pair reversed-phase high performance liquid chromatography (IP-RP-HPLC) method (stability assays) [28] and (2) concentrate and extract this analyte from the leached artificial seawater (ASW), through a weak anionic exchange (WAX) method preceding IP-RP-HPLC quantification (leaching assays).

## 2. Results and Discussion

### 2.1. Solubility in Water

The solubility of GAP was higher than 1000 mg/mL at 24 ± 1 in UPW and snSW, being classified according to the USP Pharmacopeia as a “very soluble” compound [29]. The high water solubility of GAP is in accordance with the reduced value (−7.02) of its *n*-octanol/water partition coefficient (logK_ow_) previously calculated with EPISUITE [24]. This suggests that water will retain the compound in solution, reinforcing the low affinity of GAP for sediments and fatty tissues, which makes GAP an eco-friendly candidate to be incorporated in coatings [22,24].

### 2.2. Optimization of the IP-RP-HPLC Method

An IP-RP-HPLC method was developed to quantify GAP in UPW and snSW. First, several mobile phases containing different proportions of acetonitrile and acidified water (0.1% acetic acid) were investigated. However, the retention factor (k) was not satisfactory (less than 1), with GAP overlapping with the solvent front. As the retention of this charged molecule was not achieved by regular reversed-phase, a mobile phase containing an aqueous solution of 25 mM of tetrabutylammonium bromide (TBA-Br) and acetonitrile (38:62 *v*/*v*) was used to favor ion pairing, and consequently suitable k values between 1-10 could be reached [28]. The ion pairing of GAP dissolved in UPW was accomplished, with an acceptable k value of 1. However, in snSW a k of 0 was still observed, even using different proportions of the mobile phase. In order to assure retention (k ≠ 0), using the same aqueous mobile phase containing TBA-Br and acetonitrile (38:62 *v*/*v*), a pre-treatment of the sample was carried out before injection, diluting GAP dissolved in snSW with 60 mM of TBA-Br in a proportion of 1:3, leading to ion pairing with a desirable k value of one.

### 2.3. Stability of Sulfate Groups in Water

To assess the stability of GAP in water, its half-life (DT_50_) was evaluated in UPW and snSW. GAP was exposed to several stress conditions (4 °C, 18 °C and r.t. (24 ± 1) in the absence and presence of natural light) in order to mimic different natural environmental conditions and its stability was determined at 0, 2 and 9 months.

**Figure 2:**
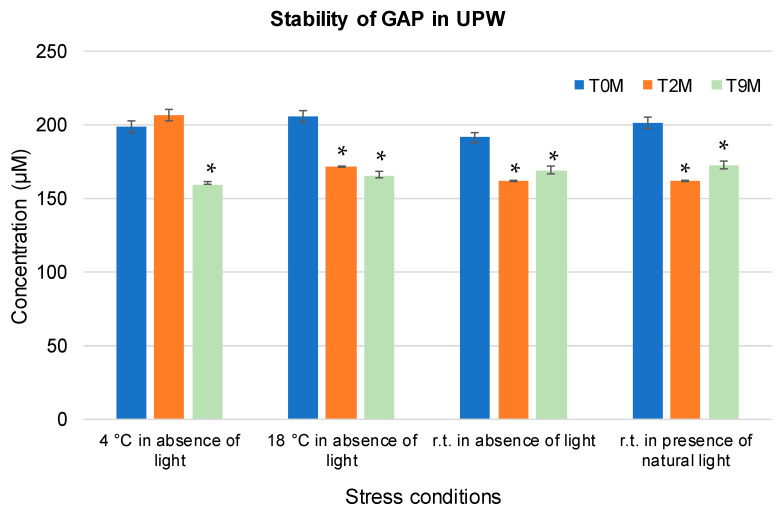
Stability of gallic acid persulfate (GAP) dissolved in ultra-pure water (UPW) (200 µM) and exposed to several stress conditions over a period of 9 months. * Indicates significant differences at *p* < 0.05 (Dunnett test) against the negative control (initial time (T0M), blue bar). T0M: initial time; T2M: two months; T9M: nine months).

In UPW, the concentration of GAP significantly decreases after the first 2 months (except at 4 °C in darkness), but it remains approximately constant during the following consecutive months, for a total of 9 months (Figure 2), suggesting the chemical stability of the sulfate linkage (without significant differences (*p* < 0.05) between the initial time and the ninth month).

**Figure 3:**
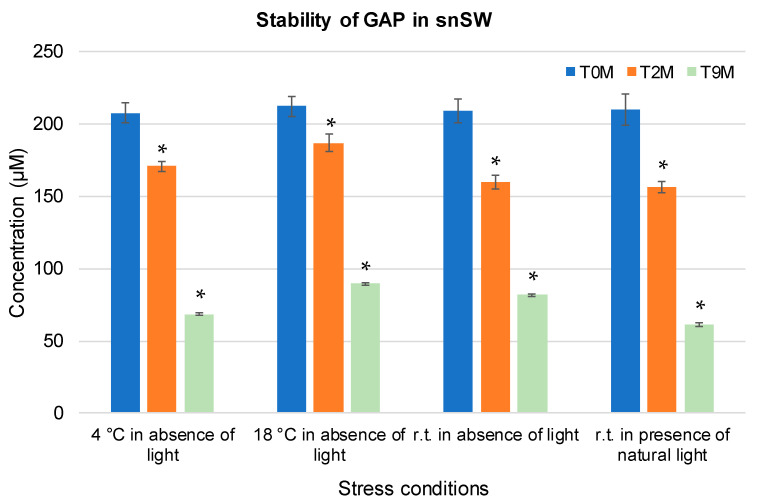
Stability of gallic acid persulfate (GAP) dissolved in sterilized natural seawater (snSW) (200 µM), exposed to the several stress conditions over a period of 9 months. * Indicates significant differences at *p* < 0.05 (Dunnett test) against the negative control (T0M, blue bar). T0M: initial time; T2M: two months; T9M: nine months).

After 9 months in snSW, GAP was abiotically degraded, reaching a significant 50% degradation (*p* < 0.05) from which a half-life (DT_50_) value of 7 months in all tested stress conditions was calculated (Figure 3). This study also suggests that photolysis may not accelerate GAP degradation, since the degradation rate did not increase in the presence of light and there are non-significant differences (*p* < 0.05). In the future, the identification of transformation products by liquid chromatography associated to high resolution Mass Spectrometry (LC-MS), as well as their ecotoxicological evaluation, will be performed, providing information to regulatory authorities.

### 2.4. GAP-Based Marine Coatings

#### 2.4.1. Synthesis Optimization

**Scheme 1:**
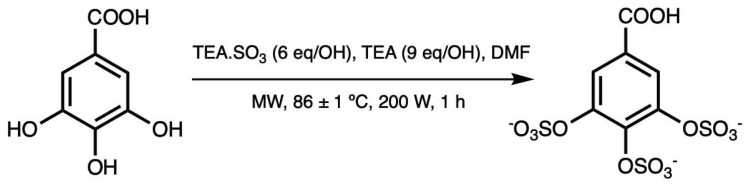
Synthesis of gallic acid persulfate (GAP).

The synthesis of GAP was optimized to obtain sufficient amounts of this product in order to prepare marine coating formulations. A reproducible and feasible one-step synthesis of GAP was accomplished by sulfation of gallic acid with the triethylamine sulfur trioxide adduct (TEA·SO_3_) and triethylamine (TEA), in dimethylformamide (DMF), under MW heating (Scheme 1). Gallic acid is a commercially available and affordable raw material and it can be obtained from several sources, such as winery waste [25].

MW irradiation is a potent green chemistry tool and, in the last decade, also emerged as a new alternative approach to obtain sulfated derivatives [30,31,32,33]. The technique offers a simple, clean, fast, efficient, and economic strategy for the synthesis of a large number of organic molecules [34]. MW heating increased the efficiency of the synthetic method, demanding lower reaction times when compared to conventional heating, and significantly increased the yield of the reaction (Table 1).

Additionally, the use of a free base in the reaction mixture allowed us to overcome the difficult persulfation of gallic acid, a tri-hydroxylated molecule. After the reaction, more triethylamine (33 eq/OH) was added to ensure the conversion of the sulfate groups into triethylamine salts, which were more easily separated in oil form in the crude product and led to an improved yield when compared to the previous purification process. The instability of the triethylamine salts was then overcome by quick conversion into sodium salts with sodium acetate. The grade of the newly synthesized GAP was similar to the grade of previous GAP (HPLC, Figure S1). The infrared and ^1^H nuclear magnetic resonance (NMR) spectra were in accordance with the literature [35].

#### 2.4.2. GAP Immobilization Strategies

To evaluate the potential of GAP as an AF agent in real coating systems, this compound was, for the first time, directly incorporated (DI) and chemically immobilized (CI) into two representative marine coatings: PDMS and PU-based coatings. The direct incorporation allows us to assess the feasibility of the new synthetized GAP as an AF agent in conventional release AF coating systems, while the chemical immobilization strategy demonstrates the potential of GAP to be grafted in polymeric non-release coating systems [36], thus promoting long-lasting effects in the generated protective coating compared with the short lifetime of the conventional releasing systems.

**Figure 4:**
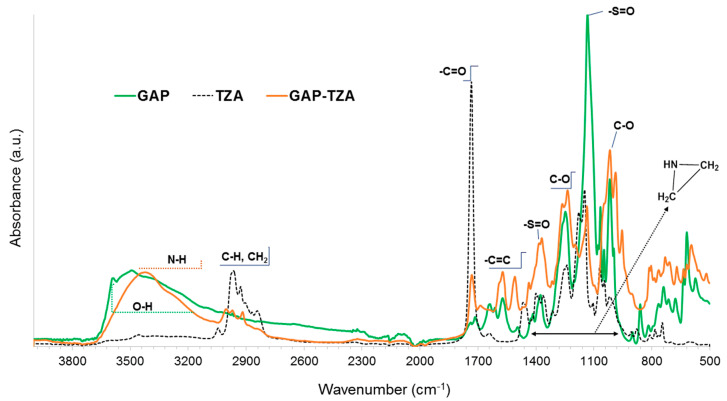
Normalized infrared spectra (FTIR-ATR) of gallic acid persulfate (GAP, green line), triaziridine propionate crosslinker (TZA, black line) and GAP–TZA derivative (brown line).

The CI GAP was promoted by the addition and blending of the trimethylolpropane triaziridine propionate crosslinker (TZA) in the coating formulations. TZA is a versatile crosslinker which reacts with functional groups of coating components carrying an active hydrogen (e.g., alkoxy), as happens with the carboxyl function in the GAP structure. The immobilization effectiveness was confirmed by analyzing the interaction of TZA with the GAP AF agent through Fourier Transform Infrared Spectroscopy (FTIR) analysis. Figure 4 shows the obtained GAP derivative (GAP–TZA) and the spectra of GAP and TZA for comparison purposes. The assignment of the spectra was carried out by calculating the vibrational spectra of GAP and TZA and comparing them to the spectra of GAP, TZA, and the GAP–TZA derivative (DFT, ADF/BP86/TZ2P; see structures and computational details in Figure S2 in Supplementary Materials).

The FTIR spectrum of GAP clearly shows the broadened characteristic band assigned to the hydroxyl stretching vibration frequencies of the carboxylic acid group, ranging from 3600 cm^−1^ to 3200 cm^−1^, with a maximum at 3494 cm^−1^. It also shows vibrational modes between 1750 cm^−1^ and 1700 cm^−1^, assigned to carbonyl stretching (C=O) of the carboxylic acid function, at 1612 cm^−1^ and 1571 cm^−1^, assigned to the aromatic C=C stretching vibrations, and from 1415 cm^−1^ to 1380 cm^−1^, assigned to S=O stretching. The bands from 1338 cm^−1^ to 1200 cm^−1^ correspond to the characteristic acyl (–C–O) stretching vibrations and can overlap with the asymmetrical stretching vibrations of the aryl sulfate. Lower frequency vibrational modes, ranging from 1010–1100 cm^−1^, are attributed to the C–O stretching vibration, and the strong band at 1134 cm^−1^ to the symmetrical stretching of aryl sulfate (S=O). Bands between 550–590 cm^−1^ and 617–650 cm^−1^ can be assigned to SO_3_ bending vibrations, and in the 757–838 cm^−1^ range to S–(OC) stretching vibrations.

The TZA spectrum shows an intense band at 1751 cm^−1^ assigned to the C=O stretching vibrational modes, corresponding to the carbonyl group of saturated aliphatic esters, and bands ranging from 1400 to 1040 cm^−1^ assigned to the stretching vibrations of aziridine rings.

**Scheme 2:**
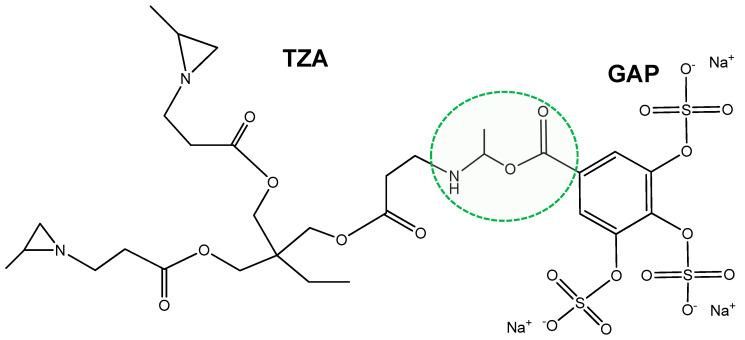
Illustration of a GAP–TZA derivative linkage obtained upon direct reaction of gallic acid persulfate (GAP) with triaziridine propionate crosslinker (TZA).

The spectrum of the GAP–TZA derivative additionally shows a distinct band at 3400 − 3300 cm^−1^, characteristic of the amine stretching vibrations, suggesting that the opening of the aziridine ring of TZA took place upon reaction with GAP. Moreover, the shift in the carbonyl stretching vibrations (from 1736 cm^−1^ in TZA and 1754 cm^−1^ in GAP) to 1732 cm^−1^ in GAP–TZA suggests the intermolecular bonding of TZA to carboxyl groups of GAP to yield amino-ester bonds (Scheme 2) [37].

^1^H and ^13^C nuclear magnetic resonance (NMR) spectra of GAP, TZA, and the GAP–TZA derivative were also obtained in DMSO-*d_6_* (c.f. SI). Despite the poor solubility of GAP–TZA and the low resolution of the spectrum, the identification of some signals reinforces the FTIR results. The chemical shifts at δ ≅ 7.50 ppm correspond to the aromatic protons of the GAP while the chemical shifts from 0.80–4.80 ppm indicate the presence of a TZA moiety, according to the ^1^H NMR of a similar compound, TMPTA-AZ [37]. In the GAP–TZA ^1^H NMR spectrum, the signal corresponding to the aromatic protons of the gallic moiety was observed at 7.52 ppm, which was more protected than in free GAP (7.85 ppm), as expected. An additional resonance observed in this region (7.37 ppm) could be attributed to a minor impurity (from reagents). Its significance may be enhanced due to the low solubility of GAP–TZA. The new signal at 1.63 ppm (^1^H NMR) and 25.91 ppm (^13^C) assigned to C14 (Figure S6, SM), as well as a low intensity signal at 2.01 ppm (NH) in the ^1^H NMR, suggest that the reaction between GAP and TZA occurred with the opening of the ring. Additionally, the ^1^H and ^13^C NMR spectra also indicate that the reaction did not occur in all three aziridine units, since it is still possible to observe signals corresponding to those units (Figures S1–S4): 1.31–1.39 ppm (H_5_), 34.25 ppm (C_5_); 1.01 ppm (H_4_), 18.70 ppm (C_4_); 1.17 and 1.29 ppm (H_6_ and H_6′_), 34.21 ppm (C_6_).

#### 2.4.3. Marine Coating Formulations

The main goal was to achieve compatible behavior between the GAP compound and the selected coating systems (DI and CI), to determine the limiting GAP content supported by each system. Several formulations of the coatings were optimized in an iterative process considering different GAP contents, solvent compatibilities, and incorporation of additional paint additives. Finally, the optimum GAP concentration was selected as the one that did not damage the main paint properties, i.e., the formulation looked the same as the control (coating system without the immobilization of GAP), Figure S7. The most promising optimized formulations are presented in Table 2 and were selected to pursue further evaluations.

#### 2.4.4. GAP Quantification in Leaching Water Samples

To evaluate the effectiveness of the CI strategy in minimizing the release of GAP from the coatings into the aquatic environment, leaching tests were performed using polyvinylchloride (PVC) plates coated with the selected marine coatings formulations. After a period of 45 days, the released GAP was extracted from leaching water samples through an optimized weak anionic exchange (WAX) methodology and then dissolved in UPW. The WAX stationary phase possesses weak groups (secondary amines), which are activated at a reduced working pH, creating a reversible interaction with strongly acidic, acidic and neutral compounds, allowing their concentration and extraction. Therefore, a low pH loading step (pH < 5) will ionize the stationary phase to facilitate the retention of GAP in the leaching water, while the remaining impurities are discarded. The elution of GAP is achieved by increasing the pH to >11, neutralizing the stationary phase. Table 3 shows the amount of detected GAP after quantification by the previously established IP-RP-HPLC method.

After a period of 45 days, the content of GAP present in the several leaching water samples, after DI in PU and PDMS coatings, was approximately 20% (Table 3). However, after CI, the percentage of GAP released from PDMS coating was only 11% (two times lower than DI in PDMS coatings), and from PU coatings 4% (almost five times lower than DI in PU coatings). These results demonstrate the high effectiveness of the chemical immobilization to retain this water-soluble compound in marine coatings, compared to the DI strategy. This phenomenon is also clearly more pronounced for a polyurethane-based matrix, which may be explained by the higher chemical compatibility of the GAP–TZA derivative functionality with a polyurethane system, associated with the intrinsic reactivity of aziridine and its possible cross-link promotion with the highly alkoxy-reactive polyurethane system, thus allowing us to reach higher agent content in the formulations (Table 3) [17].

#### 2.4.5. Anti-Settlement Behavior of GAP after Incorporation in Coatings

**Figure 5:**
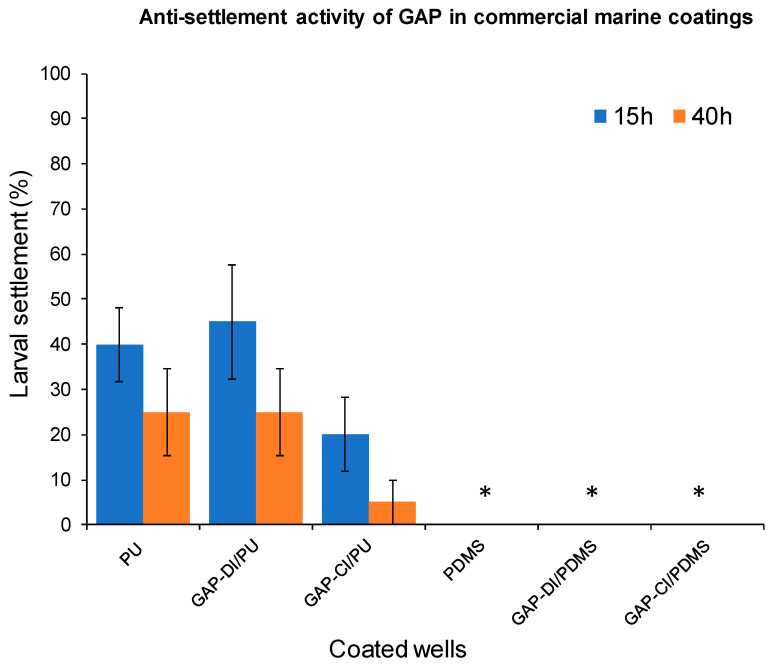
Anti-settlement activity of gallic acid persulfate (GAP) towards *Mytilus galloprovincialis* larvae after direct incorporation (DI) and chemical immobilization (CI) in commercial marine coatings. PU: polyurethane; PDMS: polydimethylsiloxane; h: hours. * No larval settlement was observed for the PDMS-based coated wells.

In order to evaluate the ability of the different GAP-containing marine coatings to prevent the attachment of mussel larvae, the anti-settlement response was evaluated at lab scale in a bioassay using *Mytilus galloprovincialis* larvae (Figure 5).

PU marine coating containing CI GAP seems to be effective against the settlement of mussel larvae, inhibiting larval settlement after both 15 h (−50%) and 40 h (−80%), in wells with this coating, compared to the control (PU). Although the individual variability of the larvae responses does not allow significantly different results against the control, the obtained settlement inhibition rates still represent a good indicator of the GAP AF potential in marine coatings. In contrast, PU marine coating containing DI GAP did not behave differently from the control. Since this conventional insoluble PU-based coating matrix acts mainly through a leaching effect [38], this result may indicate that the leaching rate and/or available GAP concentration in test media and on the outer surface of the coating is not enough to provide an effective AF action. The antifouling mechanisms involved, for instance, following leaching and/or contact strategies, would require a deep reformulation of coatings and further characterization, which go beyond the scope of this work.

In the experiments with the PDMS-based AF marine coating formulations, a high anti-settlement effect was observed in the wells coated with the control formulation, which can be explained by the coating’s intrinsic non-stick properties [39]. This experimental approach with PDMS is thus not informative regarding the efficacy of GAP, but contributes to the understanding that this matrix is, per se, highly effective against mussel larvae attachment.

Thus, to better assess the influence of the presence of GAP in the coatings’ anti-settlement properties, and also to overcome any masked effect from the intrinsic properties of the marine coatings, complementary assays with other optimized formulations of acrylic (AV) and room-temperature-vulcanizing (RTV)-PDMS-based non-marine coatings, were performed (c.f. Table 4). In the future, in situ tests will be performed to demonstrate the real potential applications of GAP-containing marine coatings in different AF systems worldwide.

**Figure 6:**
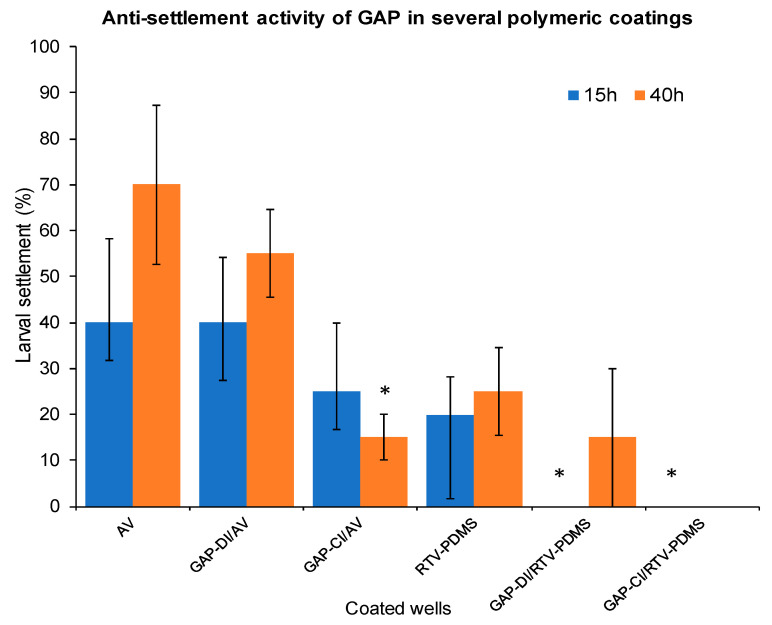
Anti-settlement activity of gallic acid persulfate (GAP) toward the macrofouling organism *Mytilus galloprovincialis larvae* after direct incorporation (DI) and chemical immobilization (CI) in conventional coatings. AV: acrylic; RTV-PDMS: room-temperature-vulcanizing polydimethylsiloxane; h: hours. * Indicates significant differences at *p* < 0.05 (Dunnett test) against the negative controls (AV and RTV-PDMS).

The anti-settlement studies with these new coatings (Figure 6) showed that, despite some decreases in larval settlement were observed in DI coatings, the CI GAP in both AV and RTV-PDMS-based formulations showed significant anti-settlement activity (*p* < 0.05), increasing the antifouling performance of both coatings even more, with only 15 and 0% of larvae adhesion being observed after 40 h of exposure, respectively.

Overall, GAP was responsible for the reduction in the settlement of a problematic marine fouling organism in different polymeric coatings after being chemically immobilized. With this optimized non-release immobilization, this very polar compound might be promising as an environment-friendly alternative resource to replace harmful commercial biocides in AF marine coatings.

## 3. Materials and Methods

### 3.1. Materials General Methods

Gallic acid was purchased from Merck (Darmstadt, Germany) (842649); triethylamine sulfur trioxide adduct (T2136) and triethylamine (TO886) were purchased from TCI (Zwijndecht, Belgium) and Sigma-Aldrich (St. Louis, MO, USA), respectively, while TBA-Br, for ion pair chromatography, was purchased from TCI (MFCD00011633, purity > 99.0%), and solvents were purchased from VWR (Radnor, PA, USA), Biosolve (Dieuze, France) and Honeywell (Seelze, Germany). snSW was collected in the Interdisciplinary Centre of Marine and Environmental Research (CIIMAR, Matosinhos, Portugal), treated with a UV filter and filtered using a 0.45-µm syringe filter. ASW was obtained from sera (sera marin salt, Heinsberg, Germany)—33 g/L of sera salt in distilled water.

MW reactions were performed in open glassware vessel reactors in a Milestone Ethos MicroSYNTH 1600 MW (Labstation, ThermoUnicam, Portugal). Thin-layer chromatography (TLC) separations were performed using Merck silica gel 60 (GF254) plates or Macherey–Nagel (Düren, Germany) octadecyl-modified HPTLC silica layers (RP-18 W/UV254). Compounds were visually detected by absorbance at 254 nm and/or 365 nm. Solvents were evaporated using a rotary evaporator under reduced pressure in a Buchi Waterbath B-480 (Flawil, Switzerland). UPW was obtained from the Milli-Q System (Millipore, Darmstadt, Germany).

Infrared spectra for the GAP and GAP–TZA derivative were obtained by FTIR analysis in a Nicolet Magna FTIR 550 Spectrometer coupled with an attenuated total reflectance unit from Smart MiracleTM-Pike Technologies (Fitchurg, WI, USA) with an individual ZnSe crystal. Analyses were performed in the frequency range of 500–4000 cm^−1^ with a 4 cm^−1^ resolution.

^1^H and ^13^C (APT) NMR spectra were recorded on a a Bruker Avance 400 spectrometer (Rheinstetten, Germany), operating at 293 K and a frequency of 400.13 MHz for ^1^H NMR, 100.61 MHz for ^13^C NMR.

IP-RP-HPLC analyses were performed on a Thermo SCIENTIFIC SpectraSYSTEM (Waltham, MA, USA) equipped with a SpectraSYSTEM UV-8000 diode array detector (DAD), a SpectraSYSTEM P4000 pump and a SpectraSYSTEM AS3000 autosampler, conducted on a Fortis BIO C18 column (Fortis Technologies, 5 µm, 250 × 4.6 mm); the used software was ChromQuest 5.0™.

### 3.2. Chromatographic Conditions

The mobile phase consisted of acetonitrile:water (62:38 *v*/*v*) containing 25 mM of TBA-Br buffer. After mixing, mobile phases were filtered through a 0.45-µm filter and degassed before use by an ultrasonic cleaner (Sonorex Digitec, Bandelin, Berlin, Germany). Chromatographic conditions were set at a constant flow rate of 1 mL/min in isocratic mode, the injection volume was 20 μL, the column was maintained at room temperature (20 ± 2), and the detection wavelength was set a 236 nm. Dissolved snSW GAP was pre-treated before injection, with a dilution of 60 mM of TBA-Br in a proportion of 1:3, allowing us to achieve ion pairing (IP) with a desirable k. This IP-RP-HPLC method was properly developed and validated according the ICH Guidance for Industry Q2 (R1) through several parameters, namely specificity/selectivity, linearity, precision, accuracy, range, limits of detection (LOD) and quantification (LOQ) (Tables S1 and S2) [40]. Concerning the leaching water samples, a different proportion of the mobile phase was used (50:50 *v*/*v*), in order to better visualize the signal of GAP without interference.

### 3.3. Preparation of Standard Solutions

GAP stock solutions from previous synthesis were prepared in UPW and snSW and stored in Eppendorf tubes at −20 °C. Two calibration curves (with three replicates) with the different water types were prepared within the range of 10–500 μM for stability and leaching assays and injected three times into the IP-RP-HPLC system with the UV detector settled at 243 nm. The standard solutions prepared in snSW were diluted with 60 mM of TBA-Br in a proportion of 1:3 prior to injection. A calibration graphic was constructed by plotting the mean peak area versus concentration.

### 3.4. Water Solubility

The water solubility was evaluated by the Shake Flask method [41]. Briefly, 6 mg of the compound was added to 6 μL of UPW and snSW in Eppendorf tubes. The tube was stirred at 24 ± 1 for 1 h and, after this time, GAP was completely solubilized in both waters. Solubility was defined according to USP [29].

### 3.5. Stability in Seawater

For the stability assay, 200 µL of stock solution was added to a total of 4 glass vials and each vial was diluted with snSW in order to obtain a final concentration of 200 µM in each vial. After that, 3 of the 4 vials were wrapped in aluminum and stored under different conditions for a period of several months: 1 vial was stored in the refrigerator at a temperature of 4 °C; 1 vial was stored at 18 °C; 1 vial was stored at 24 ± 1; and the other vial was exposed to natural light at 24 ± 1. These procedures were made in duplicate. The same assay was also performed using UPW for comparative purposes. Periodically, an aliquot of each vial was directly injected into the IP-RP-HPLC system without any extraction procedure, and the peak area of GAP was interpolated into the calibration curve.

### 3.6. Synthesis of GAP

Gallic acid (Merck 842649, 0.2 g, 1.2 mmoL) was dissolved in DMF (6 mL). Triethylamine (9 eq/OH, 3.30 mL, 32.6 mmoL) and TEA·SO_3_ (6 eq/OH, 3.8 g, 21.0 mmoL) were added. The mixture was kept under microwave radiation (200 W) at 85–87 °C, for 1 cycle of 1 h. After cooling, the mixture was held at −20 °C overnight. The inferior phase was separated and poured into acetone (100 mL). Triethylamine (12 mL, optimized proportion) was added and the mixture was left at 4 °C for a few hours. The crude oil formed was washed with acetone and ether and dissolved in aqueous solution of 30% sodium acetate (23 mL). Ethanol was added to the suspension to precipitate the sodium salt of the sulfated derivative. The yellow solid yield was 96%. The infrared and NMR data were in accordance with the literature [35]. The purity of GAP was determined by HPLC–DAD analysis with a mobile phase containing an aqueous solution of 25 mM of TBA-Br and acetonitrile (50:50 *v*/*v*) (>95%, supplementary material Figure S1). The GAP grade used for the several assays was similar (>95%), with a consistent structural characterization.

### 3.7. Immobilization of GAP in Polymeric Coatings

GAP was immobilized in two main representative biocide-free commercial marine coatings, consisting of two-component systems, a polyurethane (PU)-based system, composed of the base resin F0032 and the curing agent 95580 and a foul-release polydimethylsiloxane (PDMS) system, the HEMPASIL X3 + 87500, composed of the base resin 87509 and the curing agent 98950. Both coating systems were generously provided by Hempel A/S (Copenhagen, Denmark). The immobilization of GAP on those selected marine coating systems followed two strategies, a conventional DI and a CI. For the DI strategy, the GAP agent was first dissolved in *N*-methyl pyrrolidone (NMP, 99.5%, Acros Organics, Geel, Belgium) giving solutions with GAP contents of 18.5 and 15.9 wt.%, which were further added and blended into the paint PU and PDMS components, respectively, and in the exact amounts to yield the desirable GAP contents in the system (c.f. Table 2). The proportions based on the volume of the paint components’ bases/curing agents were 2/1 for the PU and 17.8/2.2 for the PDMS wet systems.

For the chemical immobilization strategy, a similar preparation methodology was followed, but with the additional incorporation of a trimethylolpropane triaziridine propionate crosslinker (TZA, 99.5%, PZ Global, Barcelona, Spain), in order to promote the compatibility and the grafting of GAP into the polymeric framework of the coating systems. For this strategy, GAP solutions in NMP at contents of 10.9 and 12.8 wt.% were added and blended in the PU and PDMS-based formulations, respectively. The used base and curing agent proportions for formulation preparation were the ones recommended by the supplier. Finally, the crosslinker was added and blended to the paint system in a content of 2.0 wt.% of the wet formulation.

GAP was also immobilized in two commercial non-marine coatings, an acrylic (AV) (VERKODUR, Ref. 690.195, KORELAX, Trofa, Portugal), and a room-temperature-vulcanizing polydimethylsiloxane, RTV-PDMS (RTV11, MOMENTIVE, Waterford, NY, USA). The immobilization of GAP on those selected coating systems also followed the conventional DI and CI strategies. For the first strategy, the GAP agent was previously dissolved in methyl pyrrolidone (99.5%, Acros Organics, Geel, Belgium), giving a solution containing 11.82 wt.% of GAP, which was further added and blended in the exact amount to provide the GAP-based RTV-PDMS systems (c.f. Table 4). In the case of the acrylic system, the GAP agent was directly added and blended into the acrylic components. The proportions by the volume of the paint components’ bases/curing agents were 199/1 and 3/1 for the RTV-PDMS and AV systems, respectively. For the CI strategy, a similar preparation methodology was followed with the additional incorporation of a TZA crosslinker in the blended wet formulation mixture, to promote the compatibility and the grafting of GAP into the respective polymeric matrices.

The optimized GAP contents in the developed polymeric coating formulations and additional additives were chosen in order not to compromise the main final appearance of coating films, such as apparent gloss and adhesion on the substrates used for proof-of-concept in this work, i.e., to accurately coating PVC plates for the 45-day leaching assays and the 24-well microplates for the anti-macrofouling activity assessment.

### 3.8. Evaluation of the TZA Crosslinker Interaction with GAP

In order to understand the interaction between the crosslinker and the GAP compound applied in the coating formulations to promote the GAP compatibility and its CI, a reaction between GAP and the crosslinker was performed under controlled conditions.

The TZA crosslinker was added to a three-necked round bottom flask, containing GAP (130 mg) dissolved in DMSO (1.15 mL). The mixture, with a GAP/TZA molar ratio of 1.95, was kept at room temperature, under magnetic stirring and an inert atmosphere for 24 h. A precipitate was formed, filtrated and dried in a Buchi R-210/215 rotavapor for FTIR and NMR analysis.

### 3.9. Leaching Assays

PVC-coated plates (3.5 × 6 cm) with the developed PU and PDMS polymeric coating systems, with or without immobilized GAP, were submerged in ASW for a period of 45 days, using a prior optimized stirring method [36]. The average pH of the ASW during the tests was around 8 ± 0.3, and the temperature ranged from 18 to 21 °C. The obtained leaching water samples were further collected for IP-RP-HPLC analysis.

### 3.10. Extractive Procedure

Each leaching water was divided in three portions and passed through an OASIS^®^ WAX 6cc cartridge according to the following procedure: the cartridge was conditioned with 6 mL of methanol, followed by 6 mL of water and equilibrated with 6 mL of 25 mM sodium acetate buffer acidified with acetic acid (pH 4); water samples were also acidified with sodium acetate buffer in order to obtain a pH of 4 and then passed through the cartridge; after drying, each cartridge was conditioned with 6 mL of a solution containing methanol and acetonitrile (20:80 *v*/*v*) with 2% of ammonium, followed by 2 mL of acetonitrile with 2% of ammonium, and finally 2 mL of acetonitrile; finally, the cartridge was washed with 8 mL of methanol and ammonium (90:10 *v*/*v*), followed by 2 mL of methanol basified with ammonium until a pH of 12, and the several solutions were collected in a glass vial. Solvents were reduced to dryness under nitrogen purge by a sample concentrator with a block heater at a temperature of 37 °C. After solvent evaporation, the dried compound was solubilized in 200 µL of UPW, passed through a nylon filter and injected into the IP-RP-HPLC system using a mobile phase containing an aqueous solution with 25 mM of TBA-Br and acetonitrile (50:50 *v*/*v*). Recoveries of 100% for this extractive procedure were previously obtained (Figures S8 and S9).

### 3.11. Anti-Settlement Activity Assessment of GAP Based Coatings

For a preliminary assessment of the behavior/suitability of GAP as bioactive ingredient after its incorporation in coatings, a small-scale laboratory bioassay was developed, based on the bioassay previously used to assess GAP bioactivity against mussel (*Mytilus galloprovincialis*) plantigrades [24]. For this assay, the wells of 24-well microplates were coated with the different GAP formulations to be tested (one formulation per column), and negative control columns were included for each formulation (AF agent-free coating system). The purpose of these bioassays was to determine the ability of the different GAP-based coatings to prevent the fixation of mussel larvae, testing whether the GAP bioactivity towards this macrofouling species is maintained after DI and CI procedures in different coating matrices.

Competent *Mytilus galloprovincialis* plantigrades were collected on Memory Beach (N 41°13′51.5″, W 8°43′15.5″) at low tide, and those showing exploratory behavior were selected in the laboratory and transferred to the coated 24-well microplates. All the coated wells were filled with 2.5 mL snSW (previously treated by UV light and carbon filters and mechanically filtered with 0.45 μM filter before use) to reduce any interferents in relation to mussel larvae fixation ability and health conditions. Each coating, including negative controls, was tested in four replicates (4 wells in a column) with five plantigrades per well in the darkness. After 15 and 40 h, the percentage of larval settlement was determined by the presence/absence of efficiently attached byssal threads, produced by each individual in each condition. The reading times (15 h and 40 h) for larval attachment were selected based on previous trials for bioassay optimization, where 15 h is adequate for maximum thread production and efficient attachment in normal conditions, and 40 h is the maximum time to guarantee the good health status of larvae under bioassay conditions.

### 3.12. Statistical Analysis

Data from the water stability and anti-settlement screenings were analyzed using a one-way analysis of variance (ANOVA) followed by a Dunnett test against the control (*p* < 0.05). The software IBM SPSS Statistics 21 was used for statistical analysis.

## 4. Conclusions

In this work, the synthesis of a nature-inspired antifouling compound (GAP) was optimized to maximize GAP production. GAP was obtained with the same purity as the previous methods and with better yields in only 1 h under MW irradiation. This more reproducible, feasible and greener synthesis will certainly allow an easier scale-up synthesis of GAP for future in situ studies and commercialization. MW irradiation is an established way to speed up chemical syntheses, and dominates the pharmaceutical industry, as it improves the homogeneity of the synthesis. Using a parallel scale-up approach, it is possible to achieve scalability for potential industrial adoption by using several vessels simultaneously. Furthermore, it is expected that this technology can increase the overall efficiency of organic synthesis in an environmental friendly way. On the other hand, the obtention of the raw material gallic acid, through the valorization of winery waste by a green extraction method, will also be considered in the future.

Additionally, the several analytical methods developed during this work, using ion pairing chromatography and WAX, provided important contributions to the analysis of sulfated and highly polar compounds in water.

Moreover, the potential of this very polar compound to be applied as an antifouling additive in polymeric coating systems has been proven by its successful immobilization in marine coatings, using a non-leaching strategy promoted by the incorporation of the TZA crosslinker. This triaziridine functional crosslinker reacted with the active hydrogen of the carboxyl function in the GAP structure, thus acting as a bridge molecule between the coating components and GAP. Furthermore, the GAP-based coatings seem to keep their bioactivity, at least regarding the fixation of mussel larvae. These results encourage further in situ studies with GAP as an AF additive in coatings, and an in-depth characterization of the final physical–chemical properties of coatings obtained.

## Figures and Tables

**Table 1:** Comparative conditions for sulfation of gallic acid.

Reaction	Reagents	Solvent	Reaction Conditions	Reaction Time	Yields
Previous synthesis [35]	TEA·SO_3_ (2 eq/OH)	DMA	Conventional heating 65 °C	24 h	36%
Optimized synthesis	TEA·SO_3_ (6 eq/OH) TEA (9 eq/OH)	DMF	MW 200 W 86 °C	1 h	98%

TEA·SO_3_: triethylamine-sulfur trioxide adduct; TEA: triethylamine; DMA: dimethylacetamide; DMF: dimethylformamide; h: hours; MW: microwave.

**Table 2:** Marine coating formulations with gallic acid persulfate (GAP).

Coating Formulation	Polymeric Matrix	Base/Curing Agent Ratio (*v*/*v*)	GAP Content (wt.%)	TZA Content (wt.%)
GAP-DI/PU	Polyurethane	2/1	1.99 ± 0.02	----
GAP-CI/PU	Polyurethane	2/1	2.05 ± 0.02	2.06 ± 0.02
GAP-DI/PDMS	Polydimethylsiloxane	17.8/2.2	0.55 ± 0.02	----
GAP-CI/PDMS	Polydimethylsiloxane	17.8/2.2	0.56 ± 0.02	2.05 ± 0.02

CI: chemically immobilized; DI: directly incorporated; PDMS: polydimethylsiloxane; PU: Polyurethane; TZA: triaziridine propionate crosslinker.

**Table 3:** Gallic acid persulfate (GAP) amount released from marine coatings prepared by direct incorporation (DI) and chemical immobilization (CI) strategies into artificial seawater (ASW), after an immersion period of 45 days.

Polymeric Coatings(n)	Immobilization	GAP Amount in Coated PVC Plates (mg) *	Amount of Detected GAP Leached to Waters after 45 Days (mg) *	Content of Released GAP from Coated PVC Plates (wt.%) *
GAP-DI/PU(2)	Direct	26.90 ± 2.60	4.95 ± 0.25	18.49 ± 0.86
GAP-CI/PU(2)	Chemical	34.00 ± 1.7	1.37 ± 0.06	4.02 ± 0.03
GAP-DI/PDMS(2)	Direct	6.75 ± 0.25	1.39 ± 0.02	20.59 ± 1.00
GAP-CI/PDMS(2)	Chemical	4.95 ± 0.75	0.55 ± 0.13	10.94 ± 0.90

(n): number of replicates; PDMS: polydimethylsiloxane; PU: polyurethane; PVC: polyvinyl chloride. * mean values ± standard deviation of two independent experiences.

**Table 4:** Non-marine coatings formulations with immobilized gallic acid persulfate (GAP).

Coating Formulation	Polymeric Matrix	Base/Curing Agent Ratio (*v*/*v*)	GAP Content (wt.%)	TZA Content (wt.%)
GAP-DI/AV	Acrylic	3/1	0.56 ± 0.02	----
GAP-CI/AV	Acrylic	3/1	0.56 ± 0.02	2.46 ± 0.03
GAP-DI/AV	Acrylic	3/1	1.00 ± 0.02	----
GAP-CI/AV	Acrylic	3/1	1.00 ± 0.02	2.42 ± 0.03
GAP-DI/RTV-PDMS	Polydimethylsiloxane	199/1	0.58 ± 0.02	----
GAP-CI/RTV-PDMS	Polydimethylsiloxane	199/1	0.54 ± 0.02	3.08 ± 0.03

AV: acrylic; CI: chemically immobilized; DI- directly incorporated; RTV-PDMS: room-temperature-vulcanizing- polydimethylsiloxane; TZA: triaziridine propionate crosslinker.

## Supplementary Materials

The following are available online at https://www.mdpi.com/1660-3397/18/10/489/s1, Table S1: Linear regression and sensitivity data, Table S2: Accuracy, intra- and inter-day variability (precision), Figure S1: Representative chromatograms of 200 μM of GAP obtained by conventional (green line) and new optimized synthesis (black line), Figure S2: DFT optimized structures of GAP and TZA (ADF/BP86/TZ2P), Figure S3: ^1^H NMR spectra of gallic acid persulfate (GAP, green line), triaziridine crosslinker (TZA, black line) and GAP–TZA derivative (brown line) in DMSO-*d_6_* at 293 K, Figure S4: ^13^C APT NMR spectrum of gallic acid persulfate (GAP) in DMSO-*d_6_* at 293 K, Figure S5: ^13^C APT NMR spectrum of triaziridine propionate crosslinker (TZA) in DMSO-*d_6_* at 293 K, Figure S6: ^13^C APT NMR spectrum of GAP–TZA derivative in DMSO-*d_6_* at 293 K, Figure S7: Representative PVC coated plates with commercial marine coatings, from left to right: PU (polyurethane-based control); GAP-CI/PU (PU-based coating containing chemically immobilized (CI) gallic acid persulfate, GAP; PDMS (polydimethylsiloxane-based control); and GAP-CI/PDMS (PDMS-based coating containing CI GAP. Figure S8: Representative chromatograms of 500 μM of GAP dissolved in UPW before and after being passed through the cartridge, Figure S9: Representative chromatograms of 100 μM of GAP dissolved in UPW before and after being passed through the cartridge.
