# Supplementary material for: One Step Forward towards the Development of Eco-Friendly Antifouling Coatings: Immobilization of a Sulfated Marine-Inspired Compound
Source: Mar Drugs. 2020 Sep 25;18(10):489. doi: 10.3390/md18100489 (PMC7600153; doi:10.3390/md18100489)
Supplement: Supplementary file 1 [file marinedrugs-18-00489-s001.pdf]

*Supplementary Material*

# One step forward towards the development of eco-friendly antifouling coatings: immobilization of a sulfated marine-inspired compound

***Cátia Vilas-Boas[1,2], Francisca Carvalhal[1,2], Beatriz Pereira[3], Sílvia Carvalho[4], Emília Sousa[1,2], Madalena M. Pinto[1,2], Maria José Calhorda[3], Vitor M. Vasconcelos[2,5], Joana R. Almeida[2], Elisabete R. Silva[3*] and Marta Correia-da-Silva[1,2*]***

[1] Laboratório de Química Orgânica e Farmacêutica, Departamento de Ciências Químicas, Faculdade de Farmácia, Universidade do Porto, R. Jorge de Viterbo Ferreira 228, 4050-313 Porto, Portugal; catiaboas94@gmail.com (C.V.B.); francisca.carvalhal@gmail.com (F.C.); esousa@ff.up.pt (E.S.); madalena@ff.up.pt (M.M.P.); m_correiadasilva@ff.up.pt (M.C.S.)

[2] CIIMAR – Centro Interdisciplinar de Investigação Marinha e Ambiental, Avenida General Norton de Matos, S/N, 4450-208 Matosinhos, Portugal; vmvascon@fc.up.pt (V.M.V.); joana.reis.almeida@gmail.com (J.R.A.)

[3] BioISI - Instituto de Biosistemas e Ciências Integrativas, Departamento de Química e Bioquímica, Faculdade de Ciências, Universidade de Lisboa, Campo Grande, Lisboa, 1749-016, Portugal; beatrizmmgpereira@gmail.com (B.P.); mjcalhorda@fc.ul.pt (M.J.C.); ersilva@fc.ul.pt (E.R.S.)

[4] CQB - Centro de Química e Bioquímica, Departamento de Química e Bioquímica, Faculdade de Ciências, Universidade de Lisboa, Campo Grande, Lisboa, 1749-016 Lisboa, Portugal; scfcarvalho@fc.ul.pt (S.C.)

[5] Departamento de Biologia, Faculdade de Ciências, Universidade do Porto, Rua do Campo Alegre S/N, 4169-007 Porto, Portugal

* Correspondence: ersilva@fc.ul.pt (E.R.S); m_correiadasilva@ff.up.pt (M.C.S)

**Index**

Table S1. Linear regression and sensitivity data…………………………………………………………………......3

Table S2. Accuracy, intra- and inter-day variability (precision)………………...…….……………...……..4

Figure S1. Representative chromatograms of 200 μM of GAT obtained by conventional (green line) and new optimized synthesis (black line) ………………………………………………………………………………5

Figure S2. DFT optimized structures of GAP and TZA (ADF/BP86/TZ2P)……………………………………6

Figure S3. $^{1}$H NMR spectra of gallic acid persulfate (GAT, green line), triaziridine crosslinker (TZA, black line) and GAP-TZA derivative (brown line) in DMSO-$d_6$ at 293 K..……….…………………………..…… ... 8

Figure S4. $^{13}$C APT NMR spectrum of gallic acid trisulfate (GAP) in DMSO-$d_6$ at 293 K…..................... ....9

Figure S5. $^{13}$C APT NMR spectrum of triaziridine propionate crosslinker (TZA) in DMSO-d6 at 293 K..……….………………………………………………………………………………………………..…… 10

Figure S6. $^{13}$C APT NMR spectrum of GAP-TZA derivative in DMSO-d6 at 293 K..………………………...11

Figure S7. Representative PVC coated plates with commercial marine coatings, from left to right: PU (polyurethane-based control); GAP-CI/PU (PU-based coating containing chemically immobilized (CI) gallic acid persulfate, GAP; PDMS (polydimethylsiloxane-based control); and GAP-CI/PDMS (PDMS-based coating containing CI GAP. …………… ……………………………………………………..…..12

Figure S8. Representative chromatograms of 500 μM of GAT dissolved in UPW before and after being passed through the WAX cartridge………………...……………………………………………………...13

Figure S9. Representative chromatograms of 100 μM of GAT dissolved in UPW before and after being passed through the WAX cartridge…………………………………………………………….……... ….14

**IP-RP-HPLC Method validation**

The method developed to analyze a sulfated and polar compound in water was validated according to the International Council for Harmonisation (ICH) Guidance for Industry for parameters such as selectivity, linearity, range, accuracy, and precision [1].

To evaluate selectivity, several concentrations in the range of 5–300 % of the expected working range were prepared in both sample matrices (UPW and snSW), and their chromatographic signals analyzed under the same conditions (retention time and resolution), as well as spiked and non-spiked blank samples for comparative purposes. Standard snSW samples were pre-treated before injection, with a dilution of 60 mM of TBA-Br in a proportion of 1:3. Linearity was determined by calculation of a regression line from the peak area vs. concentration plot for five standard solutions (10, 25, 50, 100, 200, and 500 μM), analyzed in triplicate. This parameter has a coefficient of determination (R2) higher than 0.995. The limit of detection (LOD) and limit of quantification (LOQ) of the method were calculated based on the signal-to-noise approach by comparing measured signals from standard samples with known low concentrations of each analyte with those of blank samples and establishing the signal-to-noise ratio between 3:1 for LOD and 10:1 for LOQ (Table S1).

**Table S1.** Linear regression and sensitivity data.

| Matrix | Range (μM) | Linear Regression (n=5) | $R^2$ | LOD (μM) | LOQ (μM) |
|---|---|---|---|---|---|
| UPW | 10 - 200 | [a]y = 40748x - 364255 | 0.9983 | 0.3 | 1.5 |
| snSW | 10 - 200 | [a]y = 35156x - 29155 | 0.9996 | 0.3 | 2 |

a: y, peak area. x, concentration of the standard in (μM); n: number of data points; LOD: Limits of detection; LOQ: Limits of quantitation; snSW: sterilized natural seawater; UPW: ultra-pure water.

Accuracy tests were performed in triplicate using three different known concentrations (30, 60, and 90 μM) and their percentage of recovery in both matrices were calculated. This parameter must be between 80-120 %. Relative standard deviation (RSD) values were calculated as a measure of precision. The intra-day variability (repeatability) was determined by analysing each sample within 24 h, while the inter-day variability (reproducibility) was measured on three non-consecutive days. RSD values lower than 15 % are considered acceptable (Table S2).

**Table S2.** Accuracy, intra- and inter-day variability (precision).

| Matrix | Concentration (μM) | Accuracy (%) | SD | Intra-Day Variability (RSD) | SD | Inter-Day Variability (RSD) | SD |
|---|---|---|---|---|---|---|---|
| UPW | 30 | 84 | 1.7 | 14 | 2.9 | 11 | 1.0 |
| | 60 | 89 | 1.5 | 3 | 1.1 | 3 | 1.7 |
| | 90 | 88 | 1.2 | 2 | 1.0 | 2 | 2.2 |
| snSW | 30 | 99 | 3.1 | 4 | 1.1 | 2 | 2.1 |
| | 60 | 98 | 2.5 | 1 | 0.7 | 1 | 0.5 |
| | 90 | 103 | 1.5 | 3 | 2.1 | 2 | 1 |

SD: Standard deviation; snSW: sterilized natural seawater; RSD: Relative standard deviation (%); UPW: ultra-pure water.

**Identification of gallic acid persulfate (GAP) obtained by an optimized synthesis**

The chromatogram of the obtained product presented a retention time similar to the previous synthetised GAP, allowing the identification of the new obtained product as GAP (Figure S1).

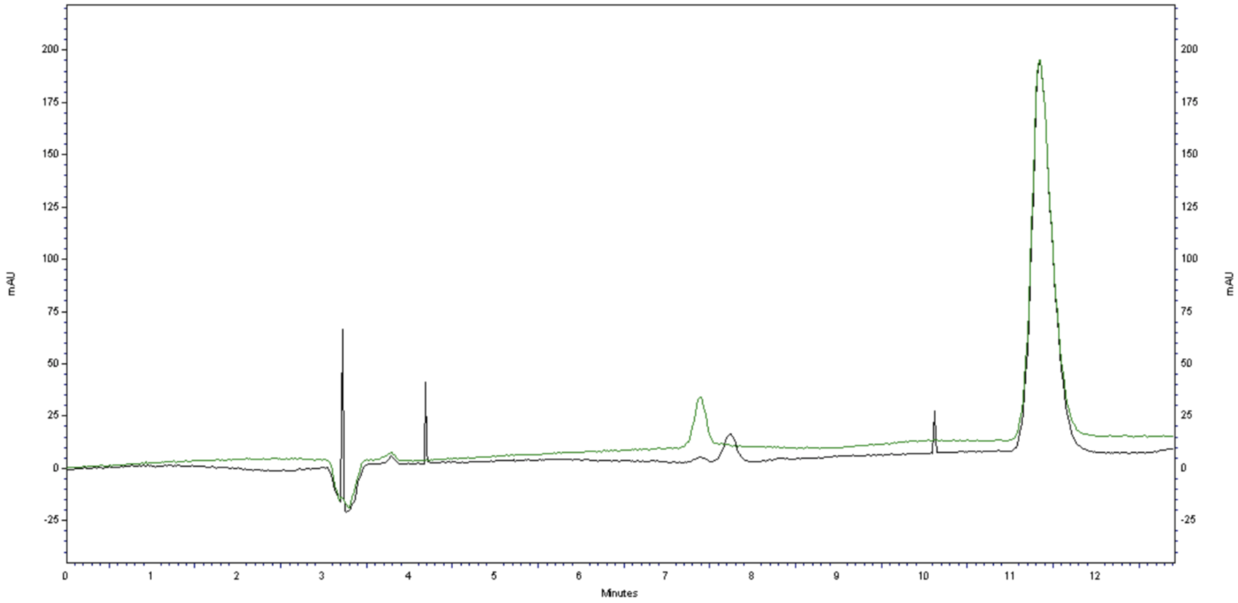


**Figure S1**. Representative chromatograms of 200 μM of GAP obtained by previous method [2] (green line) and new optimized synthesis (black line) dissolved in UPW, with a chromatographic signal at 11 min at 236 nm when a mobile phase containing an aqueous solution with 25 mM of TBAB and acetonitrile (50:50 v/v) was used.

1. ICH, Validation of Analytical Procedures: Text and Methodology. In Geneva, Q2(R1), 17 p., 2005.

2. Correia-da-Silva, M.; Sousa, E.; Duarte, B.; Marques, F.; Cunha-Ribeiro, L. M.; Pinto, M. M. Dual anticoagulant/antiplatelet persulfated small molecules. Eur. J. Med. Chem. 2011, 46, (6), 2347-58.

**Density Functional Theory (DFT) calculations.**

Density Functional Theory (DFT) calculations [1] were performed using the Amsterdam Density Functional (ADF) program [2-4]. Geometries were optimized without symmetry constraints, using the Local Density Approximation (LDA) of the correlation energy (Vosko-Wilk-Nusair) [5] and the Generalized Gradient Approximation (Becke's [6] exchange and Perdew's [7, 8] correlation functionals) with gradient correction. Relativistic effects were treated with the ZORA approximation [9]. Triple-ζ Slater-type orbitals (STOs) were used to describe all the valence electrons of H, O, C, N, and S. A set of two polarization functions was added to H (single ζ 2s, 2p), O, C, N, and S (single ζ, 3d, 4f). Frequency calculations were performed to obtain the vibrational spectra and to check that intermediates were minima in the potential energy surface. Vibrational spectra were analyzed with Chemcraft [10].

The optimized structures of GAP and triaziridine crosslinker (TZA) are represented in Figure S2.

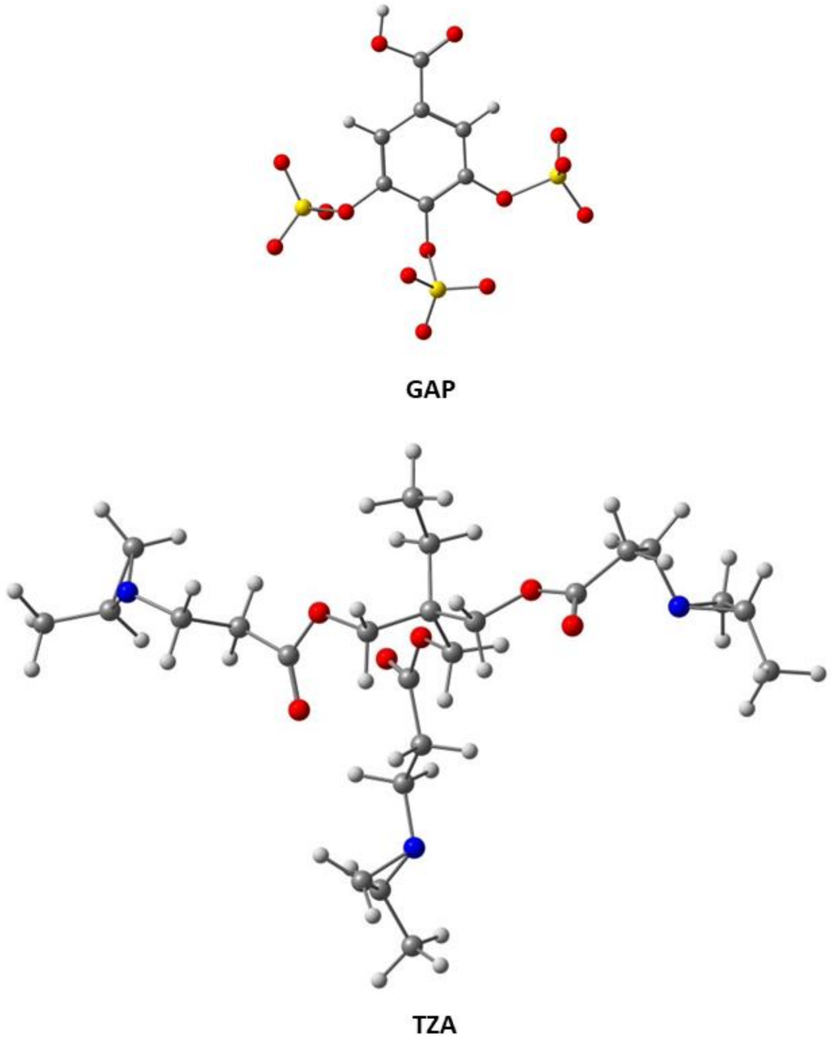


**Figure S2.** DFT optimized structures of GAP and TZA (ADF/BP86/TZ2P).

1. Parr, P. G.; Yang, W. Density-functional theory of atoms and molecules. Oxford University Press: New York, Oxford, 1989.

2. te Velde, G.; Bickelhaupt, F. M.; Baerends, E. J.; Fonseca Guerra, C.; van Gisbergen, S. J. A.; Snijders, J. G.; Ziegler, T. Chemistry with ADF. J. Comput. Chem. 2001, 22, (9), 931-967.

3. Fonseca Guerra, C.; Snijders, J. G.; te Velde, G.; Baerends, E. J. Towards an order-N DFT method. Theor. Chem. Acc. 1998, 99, (6), 391-403.

4. Theoretical Chemistry, Vrije Universiteit, Amsterdam, T. N. ADF2016 SCM Available online: http://www.scm.com (accessed 26 July 2020).

5. Vosko, S. H.; Wilk, L.; Nusair, M. Accurate spin-dependent electron liquid correlation energies for local spin density calculations: a critical analysis. Can. J. Phys. 1980, 58, (8), 1200-1211.

6. Becke, A. D. A new inhomogeneity parameter in density-functional theory. J. Chem. Phys. 1998, 109, 2092-2098.

7. Perdew, J. P. Density-functional approximation for the correlation energy of the inhomogeneous electron gas. Phys. Rev. B 1986, 33, (12), 8822-8824.

8. Perdew, J. P. Erratum: Density-functional approximation for the correlation energy of the inhomogeneous electron gas. Phys. Rev. B 1986, 34, (10), 7406-7406.

9. van Lenthe, E.; Ehlers, A.; Baerends, E.-J. Geometry optimizations in the zero order regular approximation for relativistic effects. J. Chem. Phys. 1999, 110, (18), 8943-8953.

10. Chemcraft. Available online: http://www.chemcraftprog.com/index.html (accessed on 31 July 2020).

## Nuclear Magnetic Resonance (NMR) Spectra

$^1H$ and $^{13}C$ (APT) NMR spectra were recorded on Bruker Avance 400 spectrometer, operating at 293 K and a frequency of 400.13 MHz for $^1H$ NMR, 100.61 MHz for $^{13}C$ NMR. The $^1H$ NMR spectra of gallic acid persulfate (GAP), triaziridine propionate crosslinker TZA, and GAP-TZA derivative are shown in Figure S2 (page 6). The $^{13}C$ (APT) NMR spectra of GAP and TZA are shown in Figure S3 (page 7) and Figure S4 (page 8), respectively, and the $^{13}C$ (APT) NMR spectrum of the GAP-TZA derivative is shown in Figure 5 (page 9).

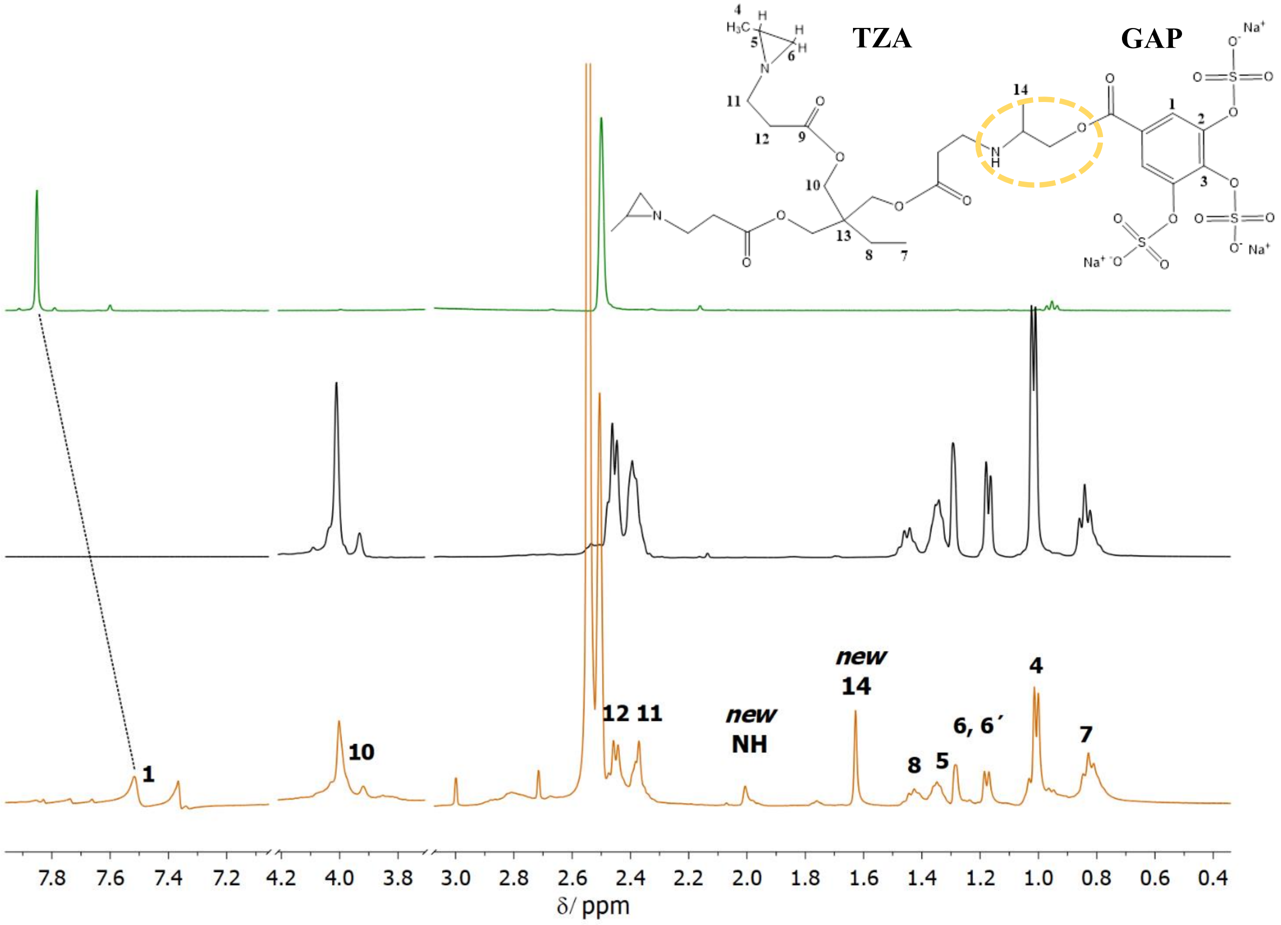


**Figure S3.** $^1H$ NMR spectra of gallic acid persulfate (GAP, green line), triaziridine crosslinker (TZA, black line) and GAP-TZA derivative (brown line) in DMSO-$d_6$ at 293 K.

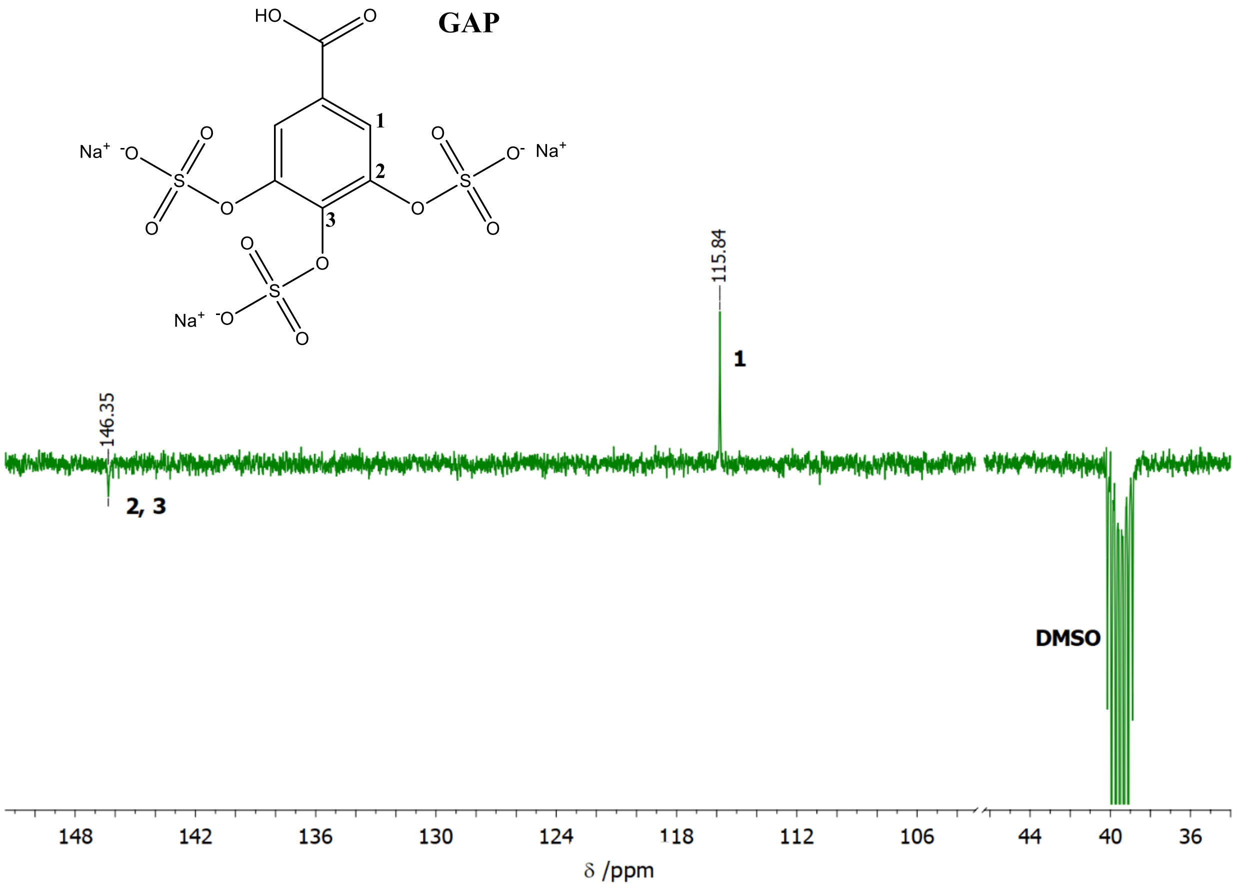


**Figure S4.** $^{13}C$ APT NMR spectrum of gallic acid persulfate (GAP) in DMSO-$d_6$ at 293 K.

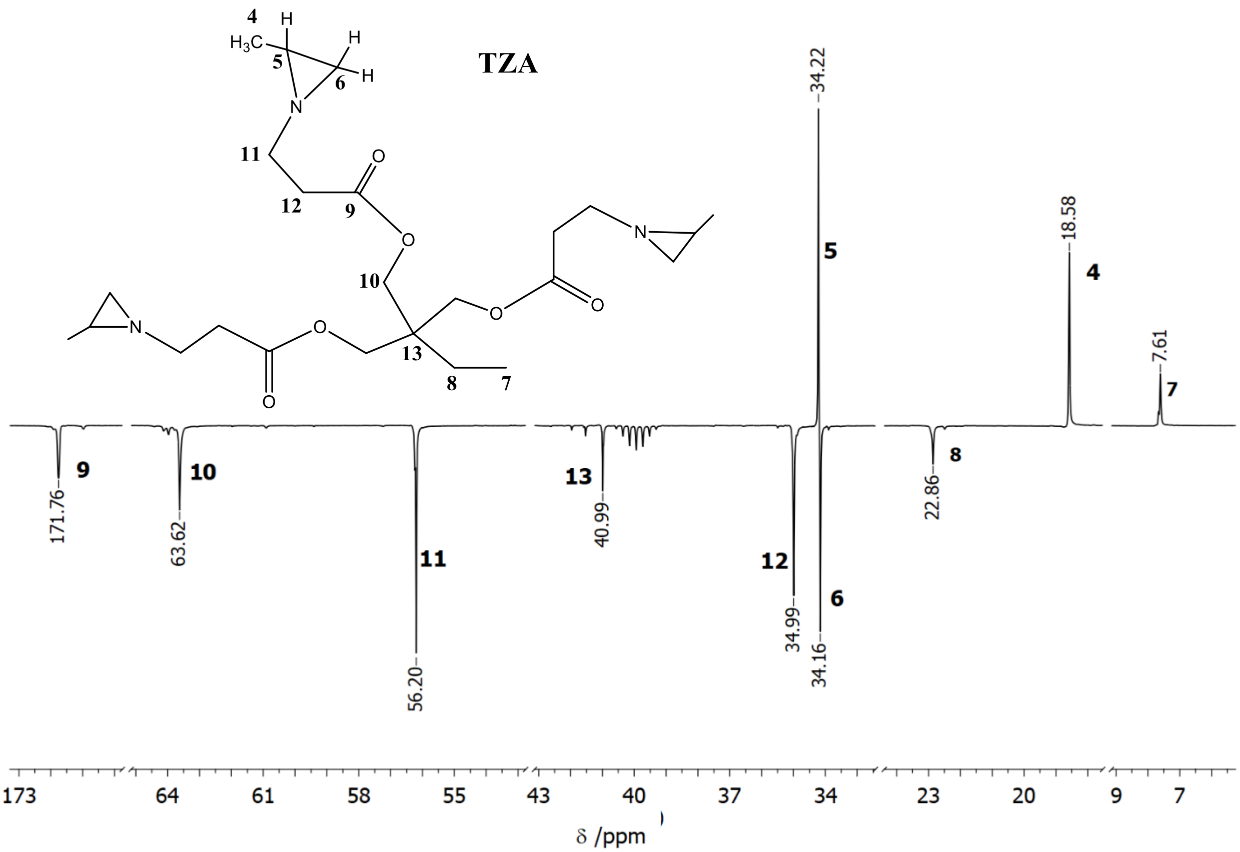


**Figure S5.** $^{13}C$ APT NMR spectrum of triaziridine propionate crosslinker (TZA) in DMSO-$d_6$ at 293 K.

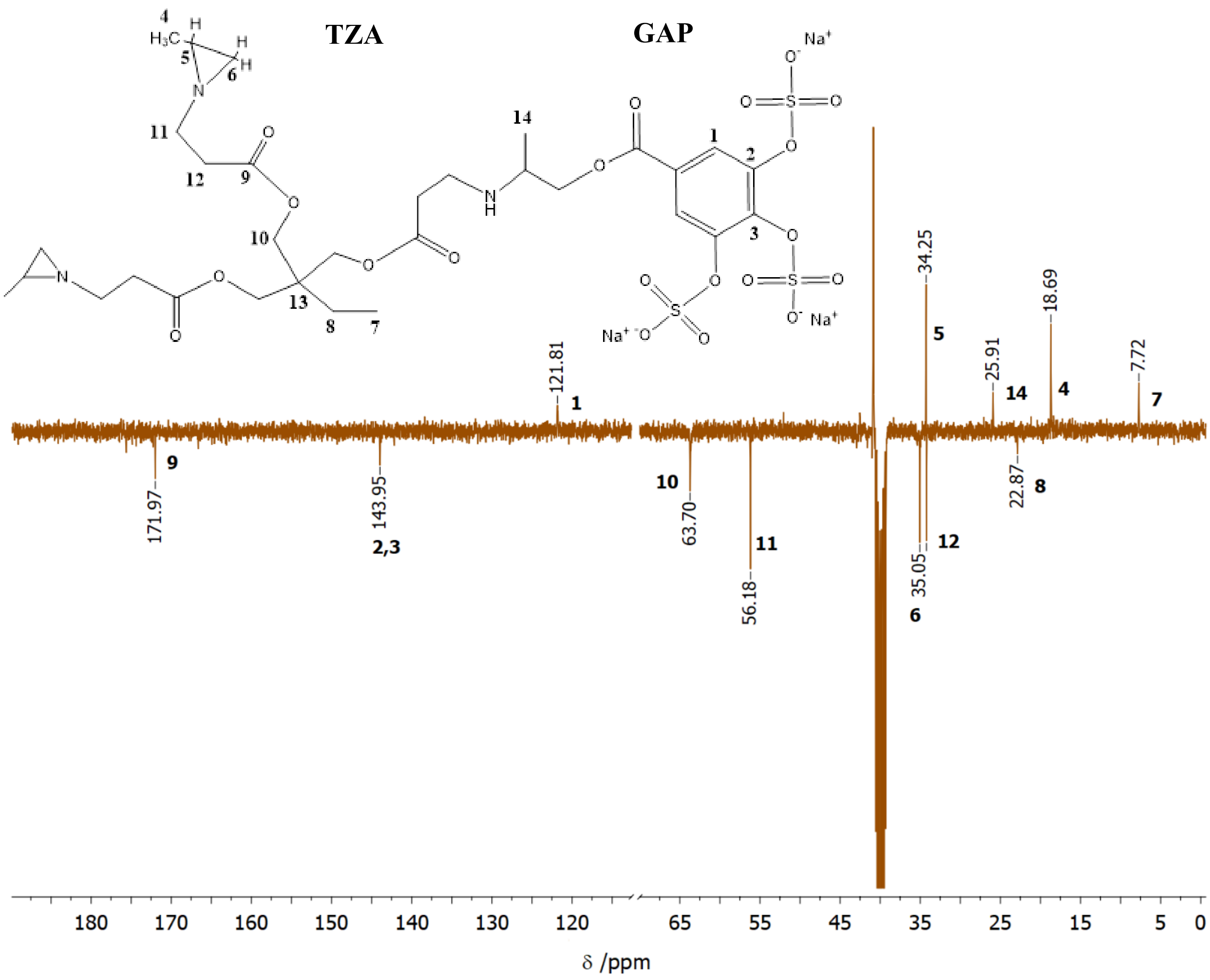


**Figure S6.** $^{13}C$ APT NMR spectrum of GAP-TZA derivative in DMSO-$d_6$ at 293 K.

**Representative PVC plates coated with gallic acid persulfate (GAP) based marine coatings and respective controls.**

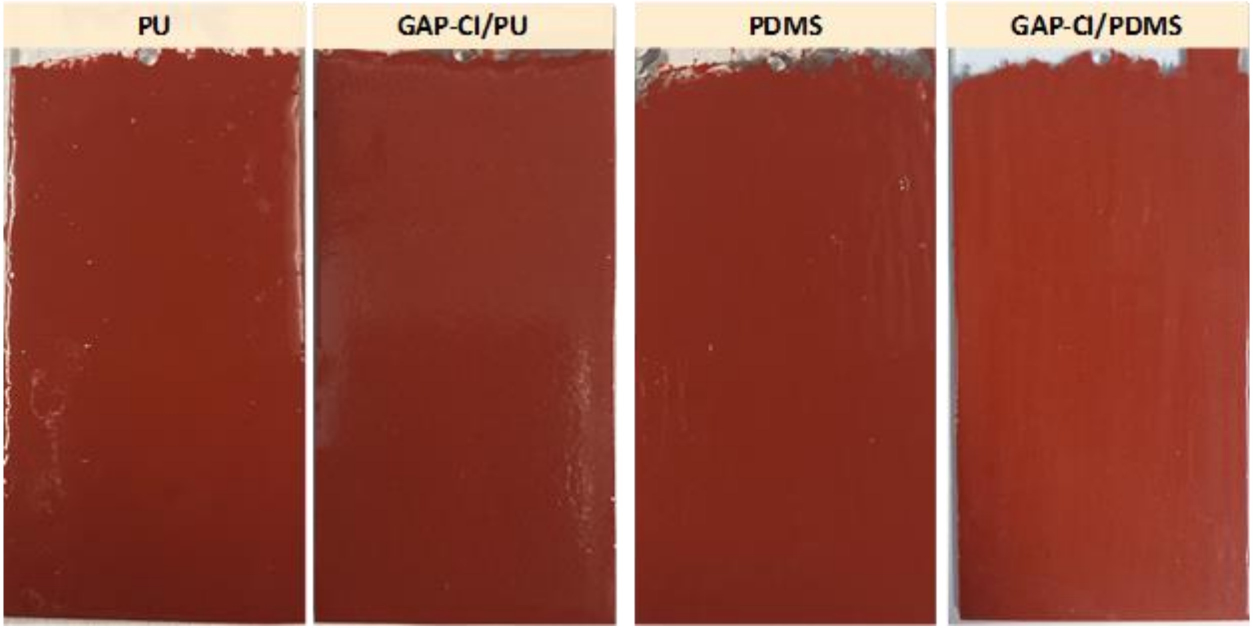


**Figure S7.** Representative PVC coated plates with commercial marine coatings, from left to right: PU (polyurethane-based control); GAP-CI/PU (PU-based coating containing chemically immobilized (CI) gallic acid persulfate, GAP; PDMS (polydimethylsiloxane-based control); and GAP-CI/PDMS (PDMS-based coating containing CI GAP.

**Recovery of GAP**

To analyze GAP recovered by the new extractive procedure using WAX cartridges, 300 µL of UPW containing 100 µM and 500 µM of GAP were dissolved in 10 mL of ASW and passed through the cartridges and subsequently extracted according the procedure described in section 2.10. The recovery rate of the extractive process was determined by comparing chromatographic signals of chromatograms with those resulting from the injection of the standard solutions before extraction. A recovery rate of 104 ± 2 % was obtained (Figure S8 and Figure S9).

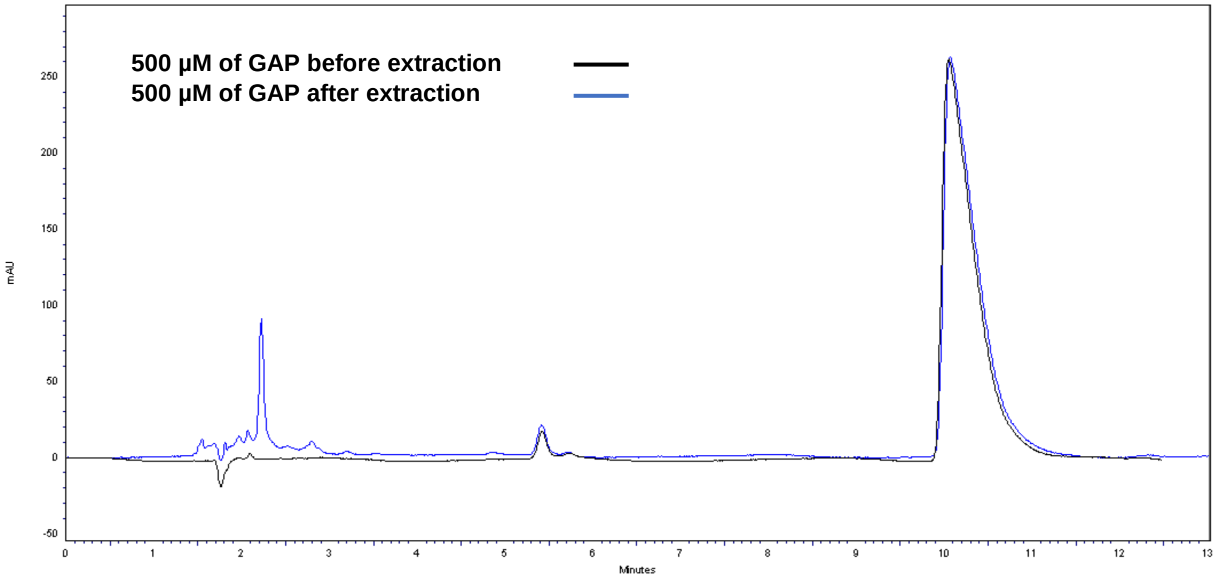


**Figure S8.** Representative chromatograms of 500 µM of GAP dissolved in UPW before and after being passed through the WAX cartridge, with a chromatographic signal at 10 min at 236 nm when a mobile phase containing an aqueous solution with 25 mM of TBAB and acetonitrile (50:50 v/v) was used.

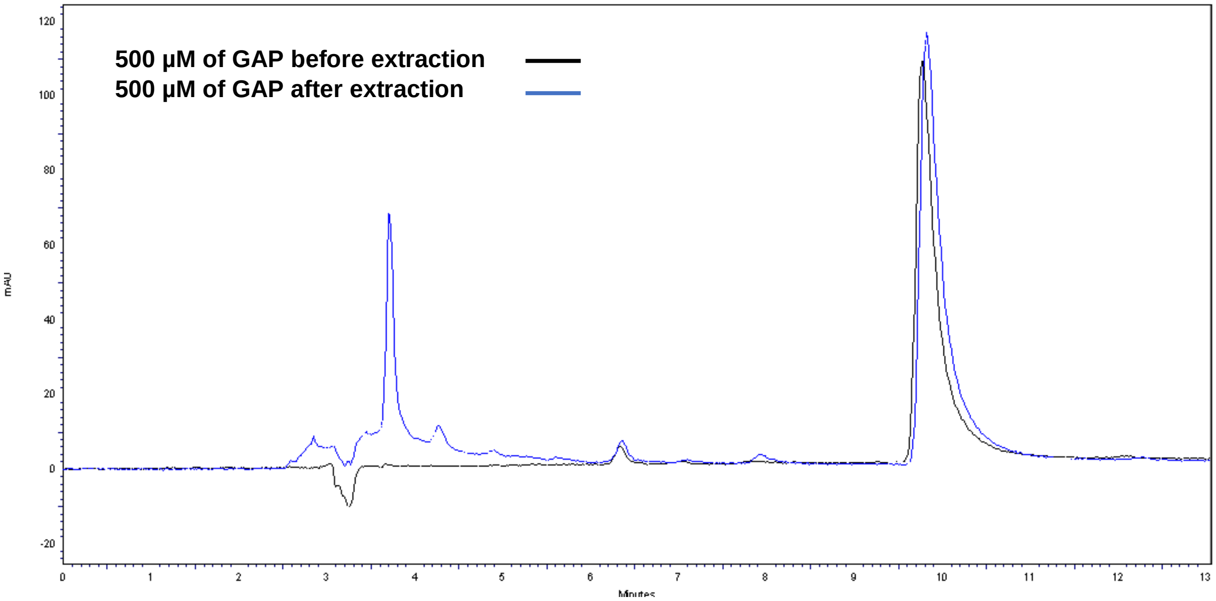


**Figure S9.** Representative chromatograms of 100 µM of GAP dissolved in UPW before and after being passed through the WAX cartridge, with a chromatographic signal at 10 min at 236 nm when a mobile phase containing an aqueous solution with 25 mM of TBAB and acetonitrile (50:50 v/v) was used.
